# Development and Application of CRISPR-Cas Based Tools

**DOI:** 10.3389/fcell.2022.834646

**Published:** 2022-04-04

**Authors:** Yanping Hu, Wei Li

**Affiliations:** ^1^ State Key Laboratory of Stem Cell and Reproductive Biology, Institute of Zoology, Chinese Academy of Sciences, Beijing, China; ^2^ University of Chinese Academy of Sciences, Beijing, China; ^3^ Institute for Stem Cell and Regenerative Medicine, Chinese Academy of Sciences, Beijing, China; ^4^ Bejing Institute for Stem Cell and Regenerative Medicine, Beijing, China; ^5^ HIT Center for Life Sciences, Harbin Institute of Technology, Harbin, China

**Keywords:** CRISPR toolkits, gene editing, Cas9, Cas12, Cas13

## Abstract

Abundant CRISPR-Cas systems in nature provide us with unlimited valuable resources to develop a variety of versatile tools, which are powerful weapons in biological discovery and disease treatment. Here, we systematically review the development of CRISPR-Cas based tools from DNA nuclease to RNA nuclease, from nuclease dependent-tools to nucleic acid recognition dependent-tools. Also, considering the limitations and challenges of current CRISPR-Cas based tools, we discuss the potential directions for development of novel CRISPR toolkits in the future.

## Introduction

As an important RNA-guided adaptive immune system in bacteria and archaea, the function of the clustered regularly interspaced short palindromic repeats-CRISPR-associated (CRISPR-Cas) system is to defend against mobile genetic elements (MGEs), including viruses, plasmids, and transposons (Sorek et al., 2013; Faure et al., 2019; Koonin and Makarova, 2019; Makarova et al., 2019). The CRISPR loci consist of Cas genes and CRISPR arrays. The function of the CRISPR-Cas system is mainly divided into three stages. First is the adaptation stage, where Cas proteins such as Cas1 and Cas2 insert foreign protospacer sequences into the CRISPR array so that it becomes a new spacer. The second is the expression stage, where the CRISPR array is transcribed into pre-CRISPR RNA (crRNA) and then processed into mature crRNA. Finally, in the interference stage, crRNA guides CRISPR effector proteins to cleave foreign target sequences such as viruses and plasmids (Barrangou et al., 2007; Brouns et al., 2008). The CRISPR system was previously thought to exist only in bacteria and archaea. However, it was recently discovered in huge phages that the CRISPR system lacks Cas proteins required for the adaptation stage, such as Cas1, Cas2, and Cas4, and the corresponding effector proteins also have gene editing capabilities (Al-Shayeb et al., 2020; Pausch et al., 2020). These CRISPR-Cas systems may target host genome to regulate host gene expression and enhance the viability of bacteriophages (Al-Shayeb et al., 2020).

The CRISPR-Cas system is competing with MGEs, which promote the evolution of the CRISPR-Cas system and greatly increase its diversity (Koonin and Makarova, 2019). The current CRISPR-Cas system is divided into class 1 and class 2 according to the effector modules (Makarova et al., 2015). The class 1 systems have effector modules composed of multiple Cas proteins and include three types and 16 subtypes, while the class 2 systems encompass a large protein and include three types and 17 subtypes (Makarova et al., 2019).

**FIGURE 1:**
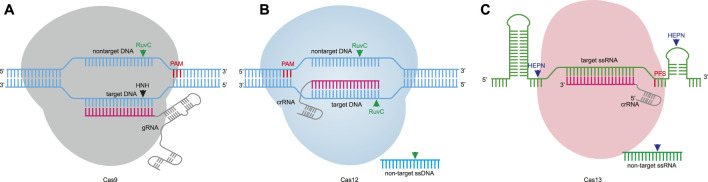
Nuclease-mediated editing. **(A)**. Cas9 nuclease is guided by gRNA for targeting specificity. The protospacer adjacent motif (PAM) sequence is located 3′ of the nontarget DNA. The Cas9 nuclease domains, RuvC and HNH, cleave the nontarget DNA and target DNA to induce predominantly a blunt DSB. **(B)**. Cas12 nuclease recognizes DNA target sequences with crRNA. The PAM motif is located 5′ of the nontarget DNA, and the RuvC-like nuclease domain cleaves both strands of DNA to generate a staggered DSB. In parallel, a target DNA can activate collateral cleavage activity to cleave non-target ssDNA through correct base-pairing to the crRNA. **(C)**. Cas13 proteins are crRNA-guided RNA-targeting nucleases, some of which require protospacer flanking sequence (PFS). The two HEPN domains cleave the target ssRNA. Analogous to Cas12, Cas13 also exhibits collateral cleavage activity to cleave non-target ssRNA.

The CRISPR-Cas system has been developed into a variety of editing tools in the past decade. Due to the complexity of class 1 members, fewer gene editing tools have been developed (Özcan et al., 2021; Dolan et al., 2019; Cameron et al., 2019). Currently, class 2 members are being developed into lots of gene editing tools. The class 2 system is divided into three types, including type II, type V, and type VI, and the corresponding effector proteins are Cas9 (Jinek et al., 2012; Jinek et al., 2013; Cong et al., 2013; Mali et al., 2013), Cas12 (Zetsche et al., 2015a; Teng et al., 2018), and Cas13 (Abudayyeh et al., 2016; Konermann et al., 2018), respectively. The class 2 systems are classified into DNA-targeting effectors such as Cas9 and Cas12, and RNA-targeting CRISPR effectors such as Cas13 (Figure 1). DNA-targeting effector proteins contain the RuvC-like domain, and Cas12 only needs the RuvC-like domain to cleave double stranded DNA (dsDNA) (Figure 1B) (Dong et al., 2016). In order to cleave dsDNA, Cas9 also requires the HNH domain. The RuvC-like domain cleaves nontarget DNA, whereas the HNH domain cleaves target DNA (Figure 1A) (Nishimasu et al., 2014). Cas13 contains two HEPN domains that are critical for the cleavage of target single-stranded RNA (ssRNA) (Figure 1C) (Liu et al., 2017a). According to the nucleic acid cleavage capability and nucleic acid binding capability of class 2 proteins, different gene editing tools can be developed.

## RNA-Guided DNA-Targeting Effectors

### CRISPR Toolkits Based on DNA Nuclease

Cas9 and Cas12 are RNA-guided DNA-targeting nucleases, which can be recruited by guide-RNA (gRNA) to cleave a specific dsDNA sequence (Figure 1). Cas12 nucleases typically generate staggered cuts, while Cas9 nucleases usually produce blunt-end cuts. Gene editing is achieved by repairing break sites through DNA repair pathways such as nonhomologous end joining (NHEJ) and homology-directed repair (HDR) (O’Driscoll and Jeggo, 2006; Gallagher and Haber, 2018). NHEJ can introduce insertions or deletions at the cleavage site and is generally used for gene knockout due to unintended insertions/deletions (indels). The HDR repair and donor sequence with homology arms can achieve accurate gene editing, which can be used for gene replacement or gene knock-in (Platt et al., 2014; Devkota, 2018).

After nearly 10 years of development, gene editing based on CRISPR technology has a very wide range of applications. In biological research, CRISPR gene editing tools are chosen for preparing mammalian models due to their simplicity, efficiency, and accuracy (Sander and Joung, 2014). Knock-in or knock-out animal models can be generated by gene editing of germline stem cells using the HDR or NHEJ repair pathways (Hsu et al., 2014). Scientists currently use CRISPR-Cas9 technology to develop animal models such as those using mice (Shalem et al., 2014), rats (Li et al., 2013a; Li et al., 2013b), pigs (Niu et al., 2017), and monkeys (Zhang et al., 2018). Because of the high efficiency of the Cas9 gene tool, it is possible to edit multiple targets in parallel and to identify potentially important genes through genome-wide functional screens. The use of a genome-scale lentiviral gRNA library can perturb thousands of genes in parallel. Researchers used a pool of single-guide RNA (sgRNA) expressing lentiviruses to infect cells and generated a large number of knockout cells after positive and negative screening, which can be used to identify important genes and drug targets (Shalem et al., 2014; Wang et al., 2014; Zhou et al., 2014; Manguso et al., 2017). Using knockout library screens, researchers successfully identified host genes that are critical to the toxicity of anthrax and diphtheria toxins, which were confirmed by functional verification (Zhou et al., 2014). Ptpn2 was identified as a cancer immunotherapy target by specifically knocking out 2,368 genes in melanoma cells (Manguso et al., 2017). In agriculture, the rise of gene editing technology has enabled greater possibilities for cultivating new varieties (Shan et al., 2013). The construction of new genetically modified crops through gene editing can increase crop quality (Xu et al., 2016). Glyphosate-resistant rice was successfully cultivated by replacing the 5-enolpyruvylshikimate-3-phosphate synthase (EPSPS) gene with Cas9 (Manguso et al., 2017). In terms of clinical application, Cas9 has been used to knock out the CCR5 gene in HSPCs for transplantation and to treat patients with HIV and acute lymphoblastic leukemia (Xu et al., 2019). Cas9 can be used to efficiently construct CAR-T cells and can be used for immunotherapy (Liu et al., 2017b). T cells knocked out of the PD-1 gene can be used to treat metastatic non-small cell lung cancer (Cyranoski, 2016). CRISPR Cas will become a promising and feasible strategy for the treatment of ophthalmic diseases in the near future (Moore et al., 2018). Recovery of normal CEP290 expression by removing abnormal splicing donors created by mutations in the CEP290 gene IVS26 is the treatment for Leber congenital amaurosis type 10 (LCA10) in mice and non-human primate (NHP) models (Maeder et al., 2019).

Unlike Cas9, Cas12 can cleave the target sequence (cis cleavage), and also activate its ability to cleave non-target sequences (trans cleavage or collateral cleavage) when the target sequence and the corresponding gRNA exist (Figure 1B) (Chen et al., 2018). Because of the trans-cleavage activity of Cas12, it can be developed into nucleic acid detection tools for detecting the presence of target sequences (Gootenberg et al., 2018; Teng et al., 2019). Cas12 was developed into DNA endonuclease-targeted CRISPR transreporter (DETECTR), which can detect the presence of single-stranded DNA (ssDNA) (Chen et al., 2018). Combined with isothermal pre-amplification, it has been proven that DETECTR quickly and accurately detects clinically relevant types of human papillomavirus (Chen et al., 2018). The nucleic acid detection tools developed by Cas12 can be applied to detect DNA viruses in clinical research, setting the stage for rapid pathogen detection (Knott and Doudna, 2018).

### CRISPR Toolkits Based on DNA-Binding Domain

**FIGURE 2:**
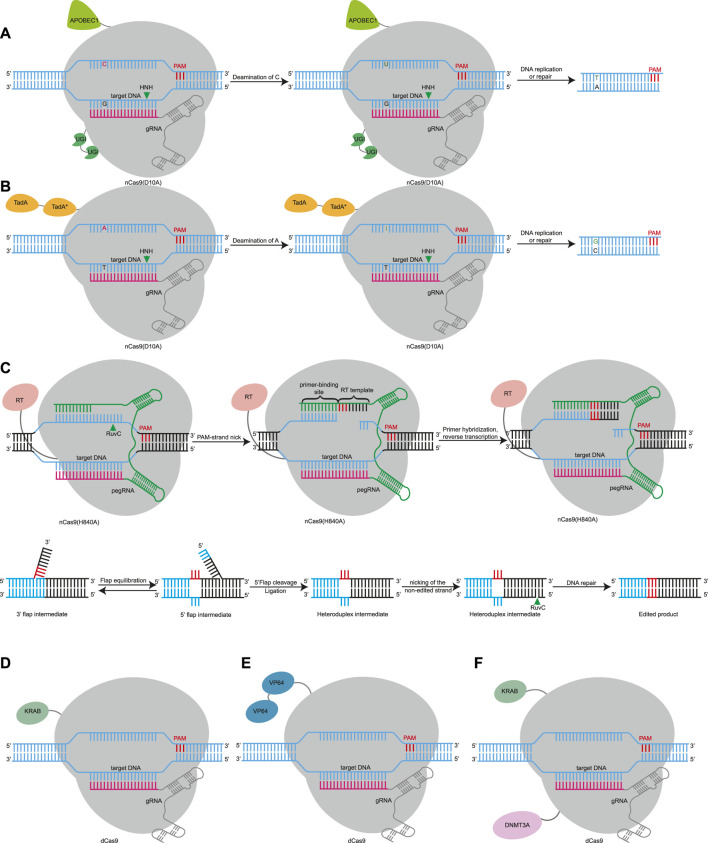
Gene editing based on RNA-guided DNA-binding domain using Cas9 effector as examples. **(A)**. CBE induces C•G to T•A point mutations using Cas9 nickase (nCas9) or dCas9 fusion with APOBEC1 and UGI. APOBEC1, cytidine deaminase enzyme. UGI, uracil glycosylase inhibitor. **(B)**. ABE uses a fusion of nCas9 or dCas9 and evolved TadA* deoxyadenosine deaminases to install A•T to G•C mutations. TadA, natural adenine deaminases. TadA*, evolved TadA. **(C)**. PE uses a reverse transcriptase fused to nCas9 and a pegRNA. The pegRNA contains a sequence complementary to the target site, which guides the PE to its target site, and a template sequence for reverse transcription. After the 3’flap of the reverse transcription product competes with the 5’flap, which is excised, and the unedited complementary strand is replaced by DNA repair using the edited strand as a template to induce insertion or deletion mutations. RT, reverse transcriptase. **(D)**. dCas9 is fused with a transcription repression domain such as KRAB can target to promoters or 5′ untranslated regions (UTRs) or enhancers to achieve more efficient interference (CRISPRi). KRAB, krüppel-associated box. **(E)**. The fusion of dCas9 with transcriptional activators such as VP64 can target promoter and enhancer regions, leading to upregulation of gene expression (CRISPRa). VP64, a strong transcriptional activation domain. **(F)**. CRISPRoff uses a fusion of dCas9 and KRAB and DNMT3A to achieve durable, heritable, and reversible DNA methylation modification and gene transcription regulation. DNMT3A, DNA methyltransferase 3 alpha.

Nuclease-dependent gene editing can induce DNA double-strand break (DSB), which may cause large disruptions. After a DSB is produced, large fragment deletions or chromosomal translocations have been reported as repair outcomes (Kosicki et al., 2018; Cullot et al., 2019; Song et al., 2020). Multiple DSBs may cause chromothripsis, activate p53-mediated DNA damage response, and even cause cancer (Haapaniemi et al., 2018; Enache et al., 2020; Leibowitz et al., 2021). Due to the insecurity of DSBs, researchers have used the characteristics of the Cas protein to develop additional gene editing tools that do not introduce DSBs. Cas9 and Cas12 possess DNA cleavage capability and DNA binding capability, and the former does not affect the latter (Jinek et al., 2012). Therefore, inactivation of either catalytic domain turns Cas9 nuclease into Cas9 nickase (nCas9) with single-strand cleavage ability, and inactivation of all catalytic domains converts Cas9 or Cas12 to deactivated Cas (dCas) without cleavage ability (Jinek et al., 2012; Zetsche et al., 2015a). These catalytically impaired Cas nucleases with DNA-binding capabilities fuse with other functional molecules such as deaminase, reverse transcriptase, transcriptional regulatory elements, or chromosome modification elements to develop additional tools (Figure 2).

A base editor (BE) consists of a DNA-modifying enzyme fused with a DNA-targeting domain, such as nCas and dCas. Fusion of cytosine deaminase and uracil glycosylase inhibitor domains (UGI) with nCas9 or dCas results in cytosine base editors (CBEs), which were developed by Liu’s team and can achieve C to T or G to A conversion (Figure 2A) (Komor et al., 2016). nCas9 directs and restricts the deaminase activity to the desired genomic site to convert cytosine nucleotides into uracil, which is read as thymine during DNA replication, DNA repair, and transcription (Komor et al., 2016). Then, they developed adenine base editors (ABEs), which can convert an AT base pair to a GC base pair (Figure 2B) (Gaudelli et al., 2017). Scientists have also developed other types of base editors (Chen et al., 2020; Kurt et al., 2020). Compared with gene editing, base editing does not cause DSBs and is more secure (Song et al., 2020). In basic biology, BEs can achieve precise editing in non-dividing cells, which can be used to construct animal models (Ryu et al., 2018), and can also edit cells with genetic defects (Nami et al., 2018). BEs can mutate codons into stop codons to knock out genes without DSBs (Billon et al., 2017). BEs can also target alternative splicing sites, leading to exon skipping (Gapinske et al., 2018). Since many genetic diseases are caused by single base mutations (single nucleotide polymorphisms, SNPs), DNA base editors are very promising for use in clinical applications. Delivery of an ABE *via* adeno-associated virus (AAV) to correct the C•G-to-T•A mutation in Hutchinson–Gilford progeria syndrome mice will prolong the lifespan of the mice by two times (Koblan et al., 2021). ABE8.8 was delivered into cynomolgus monkeys by lipid nanoparticles, introducing T to C mutations that disrupted the alternative splicing site and resulted in termination of codon production, thereby knocking down PCSK9 levels to treat high cholesterol (Musunuru et al., 2021).

The purity of the BE editing product is not high, however, and it cannot produce accurate base editing other than these four mutations. In order to solve this problem, researchers have developed prime editing (Anzalone et al., 2019). Prime editors (PEs) consist of nCas9, reverse transcriptase fusion protein, and prime-editing guide RNA (pegRNA) (Figure 2C) (Anzalone et al., 2019). PegRNA is not only responsible for guiding the PE to its target site, but it also contains the nucleotide of another newly edited sequence of RNA, which is read by reverse transcriptase and reversed into the target DNA for additional insertions or deletions. The RuvC domain of Cas9 nicks the edited strand, which contains a PAM. The primer binding sequence (PBS) of pegRNA serves as a primer, which hybridizes with the 3’ end of the nicked strand, and the reverse transcription template of pegRNA acts as a template, which contains the desired DNA alteration and the homology region of the target site. Then, pegRNA is read by RT and reversed into the target DNA for additional insertions or deletions. After reverse transcription, an edited 3’flap of the reverse transcription product competes with the original 5'flap sequence, which is subsequently cleaved and ligated. Then, nCas9 uses an sgRNA to nick the non-edited strand away from the initial nick. Finally, the nicked non-edited strand replaced by preferentially DNA repair using the edited strand as a template to induce insertion or deletion mutations (Figure 2C). It has been shown that a PE is able to generate any single base change, with at least 80 nucleotide deletions and at least 44 nucleotide insertions (Anzalone et al., 2019). In addition to gene modification by introducing stop codons or changing alternative splicing sites by the action of BEs, PEs can also introduce deletions, insertions, and any single base-to-base change without DSBs. Delivery of PE mRNA or ribonucleoprotein (RNP) into mouse or zebrafish embryos can successfully introduce the desired mutations and construct animal models (Liu et al., 2020; Park et al., 2021; Petri et al., 2021). In terms of clinical research, a PE can repair genetic mutations in sickle cell disease and Tay-Sachs disease (Anzalone et al., 2019). PEs can also achieve base replacement to treat tyrosinemia in mice (Kim et al., 2021a).

Although base editing and prime-editing technology have been widely used in disease treatment, there are many concerns, including undesirable sequences, and off-target and uncontrollable expression. Therefore, researchers fused the dCas protein with transcriptional regulatory elements or chromosomal modification elements to only regulate the level of transcription without permanently changing the genome. dCas9 targets the gene promoter region and can inhibit gene expression through steric hindrance (Qi et al., 2013). After dCas9 is fused with a transcription repression domain such as KRAB, it can achieve more efficient interference (CRISPR-mediated interference, CRISPRi) (Figure 2D) (Gilbert et al., 2013). The fusion of dCas9 with transcriptional activators such as VP64 or p65 can target promoter and enhancer regions, leading to upregulation of gene expression (CRISPR-mediated activation, CRISPRa) (Figure 2E) (Gilbert et al., 2013; Perez-Pinera et al., 2013; Maeder et al., 2013). CRISPRi and CRISPRa can be used in pooled genetic screens to identify cancer suppressor genes or functional genes (Gilbert et al., 2014), for immunotherapy tumor treatment (Wang et al., 2019a), and for reprogramming pluripotent stem cells (Liu et al., 2018). The fusion of dCas9 with epigenetic regulatory factors can achieve epigenetic regulation. The fusion of dCas9 and histone acetylation component p300 can induce high gene expression (Hilton et al., 2015). dCas9-DNMT3A can induce site-specific CpG methylation, leading to transcriptional silencing (Amabile et al., 2016). When KRAB and DNMT3A were fused to the N/C terminus of dCas9 to construct an epigenetic editor CRISPRoff (Figure 2F) (Nuñez et al., 2021), its transient expression could achieve durable, heritable, and reversible DNA methylation modification and gene transcription regulation (Figure 2F). Its application is expected in genome screening, cell engineering, enhancer silencing, and the genetic mechanism of epigenetic modification.

## RNA-Guided RNA-Targeting Effectors

### CRISPR Toolkits Based on RNA Nuclease

Cas9 and Cas12 tolerate sequence mismatches during editing, which may result in off-target (Hsu et al., 2013). Due to the persistence of genes, off-target may cause very serious consequences such as chromatin rearrangement, genomic instability, gene function disruption, and even carcinogenesis. Gene-editing tools based on RNA-targeting nuclease have been developed to temporarily edit RNA without permanently altering the DNA sequence. Cas13 is guided by crRNA and can specifically cleave targeted ssRNA (Figure 1C) (Abudayyeh et al., 2016; Cox et al., 2017; Konermann et al., 2018). Cas13 can specifically regulate gene expression levels in basic research (Abudayyeh et al., 2016; Konermann et al., 2018). LwaCas13a successfully decreased the level of mutant transcripts, but did not affect the expression of normal transcripts (Zhao et al., 2018). In clinical applications, the RNA knockdown ability of Cas13 can be used to treat viral infections. It was confirmed that Cas13 possesses potent activity against lymphocytic choriomeningitis virus (LCMV), influenza A virus (IAV), and vesicular stomatitis virus (VSV) in mammalian cells (Freije et al., 2019). Cas13d has also acts against RNA viruses in plants (Mahas et al., 2019). Cas13-based gene silencing is reversible, which has significant advantages in the treatment of acquired metabolic diseases. The knockdown of PCSK9 in mouse liver cells by Cas13d decreased blood cholesterol levels (He et al., 2020).

Analogous to Cas12, Cas13 also exhibits collateral cleavage activity, which can be used for nucleic acid detection (Figure 1C) (East-Seletsky et al., 2016). Cas13 was developed into SHERLOCK (specific high-sensitivity enzymatic reporter UnLOCKing) to detect the presence of ssRNA (Gootenberg et al., 2017; Myhrvold et al., 2018). The SHERLOCKv2 platform developed by Cas13b can simultaneously detect dengue and Zika virus ssRNA (Gootenberg et al., 2018).

### CRISPR Toolkits Based on RNA-Binding Domain

**FIGURE 3:**
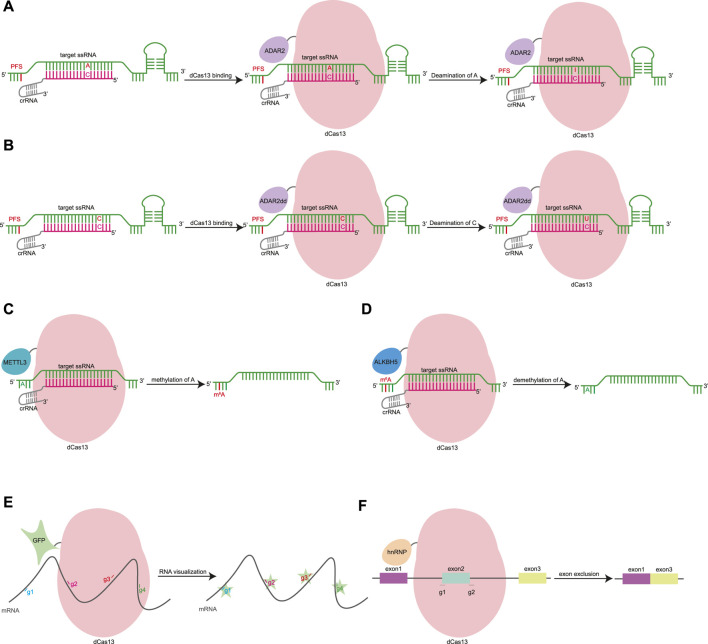
Gene editing based on RNA-guided RNA-binding domain. **(A)**. dCas13 was fused with ADAR2 to develop REPAIR, which can realize the adenosine-to-inosine conversion of RNA. ADAR2, adenosine deaminase acting on RNA type 2. **(B)**. RESCUE uses a fusion of dCas13 and evolved ADAR2dd to catalyze C-to-U conversion of RNA. ADAR2dd, evolve the adenine deaminase domain of ADAR2. **(C)**. dCas13 can be fused with truncated METTL3 methyltransferase to induce site-specific m6A changes. METTL3, methyltransferase-like 3. **(D)**. The dCas13-ALKBH5 fusion protein achieves demethylation of RNA at specific sites. ALKBH5, m6A demethylase AlkB homolog 5. **(E)**. dCas13 can be fused with GFP for mRNA imaging. g1∼4, gRNA 1–4. **(F)**. dCas13 can fuse with hnRNP, a negative regulator of splicing, to achieve targeted exon skipping. g1∼2, gRNA 1–2. hnRNP, heterogeneous nuclear ribonucleoprotein.

The HEPN domain of Cas13 can be mutated to generate dCas13, which has RNA-binding capability (Konermann et al., 2018; Cox et al., 2017). The fusion of dCas13 and RNA-modifying enzymes forms more functional tools. Cas13 can also be developed into an RNA base editor. dPspCas13b was fused with ADAR2 to develop REPAIR (RNA editing for programmable A to I replacement), which can realize the adenosine-to-inosine conversion of RNA (Figure 3A) (Cox et al., 2017). In the process of translation and splicing, inosine is read as guanine, and therefore, the REPAIR tool can restore pathogenic G-A mutations. Subsequently, the relevant team developed the RESCUE (RNA editing for specific C to U exchange) system, which not only retains the original A-I deaminase activity, but also increases the C-U deaminase capacity (Figure 3B) (Abudayyeh et al., 2019). The RNA BE can target key post-translational modification sites such as those used for phosphorylation, glycosylation, and methylation to expand the range of applications. By editing key phosphorylated residues, RESCUE inhibits ubiquitination and degradation, activates STAT and Wnt/β-catenin pathways, and promotes cell growth (Abudayyeh et al., 2019). The editing efficiency and specificity of RNA base editors are not high, and further exploration of the treatment of gene genetic diseases is necessary (Abudayyeh et al., 2019).

dCas13 can be fused with truncated METTL3 or METTL14 methyltransferase to induce site-specific m6A changes to transcript abundance and cause alternative splicing (Figure 3C) (Wilson et al., 2020). The dCas13-ALKBH5 fusion protein achieves demethylation of RNA at specific sites (Figure 3D) (Li et al., 2020). dLwaCas13 has been fused with msfGFP, nuclear localization sequence (NLS), zinc finger (ZF) protein, and the KRAB domain for mRNA imaging (Figure 3E) (Abudayyeh et al., 2017). dCas13d can fuse with hnRNP1, a negative regulator of splicing, to target pathogenic tau pre-mRNA, achieve targeted exon skipping, and decrease the ratio of dysregulated tau in a neuronal model of frontotemporal dementia (Figure 3F) (Konermann et al., 2018).

## Challenges of the Application of CRISPR‐Cas-Based Tools

Although CRISPR-Cas has so many advantages, there are still some technical challenges that must be addressed before clinical treatment of patients can proceed. Current gene editing technology can induce unintended DNA or RNA modifications. Nuclease-dependent editing may cause undesirable on-target alterations, such as large fragment deletions around DSBs or chromosomal inversions (Kosicki et al., 2018; Cullot et al., 2019; Song et al., 2020). During CRISPR-Cas editing, mismatches are tolerated during the pairing of the gRNA and the target sequence, which results in off-target (Hsu et al., 2013; Tsai et al., 2015; Kleinstiver et al., 2016a). Additionally, trans-cleavage activity of Cas12 or Cas13 may occur following the cis-cleavage of the target (Abudayyeh et al., 2016; Chen et al., 2018). In response to the harm of DSBs, researchers have developed BEs and PEs to alter sequences without introducing DSBs (Komor et al., 2016; Gaudelli et al., 2017; Anzalone et al., 2019). In order to solve the off-target problem, researchers used rational design or evolution to screen proteins with lower off-target efficiency (Kleinstiver et al., 2016b; Slaymaker et al., 2016), reduce the off-target rate by changing the composition or structure of gRNA (Zetsche et al., 2015b; Moon et al., 2019), or improve specificity by reducing the action time of the Cas protein (Kim et al., 2014). The researchers also developed the Cas7-11 editing tool without trans-cleavage activity for RNA editing (Özcan et al., 2021). The more secure CRISPR-Cas system has been the focus of future clinical applications.

Difficulties have been encountered with the *in vivo* delivery of gene editing tools. Delivery systems are divided into viral delivery and non-viral delivery. AAV, with its high infection efficiency, broad tissue tropism, and low immunogenicity, is currently recognized as the most promising and safe viral vector (Wang et al., 2019b). However, the large size of CRISPR-Cas editing tools such as spCas9, BEs, and PEs will exceed the package capability of AAV (Zhang, 2021). One strategy to solve this problem is to identify more compact Cas protein (Ran et al., 2015; Kim et al., 2017; Konermann et al., 2018; Liu et al., 2019; Yan et al., 2019; Al-Shayeb et al., 2020). The other is through non-viral delivery methods where no cargo range is involved, such as lipid nanoparticles (Musunuru et al., 2021; Rothgangl et al., 2021). The clinical application of the CRISPR-Cas system depends on the development of an appropriate delivery system.

## Perspectives

In nature, the CRISPR-Cas system has greatly increased its diversity due to the competition process with MGEs. Through the mining of metagenomic data, some new types of Cas protein with smaller size and higher security can be identified. Cas12j is a new type V system discovered in huge bacteriophages, with a protein size of 700–800 amino acids, and it has been developed as a gene editing tool (Pausch et al., 2020). Cas12f has the smallest molecular size at 400–700 amino acids (Harrington et al., 2018; Karvelis et al., 2020). Recently, researchers have developed Cas12f into a new gene editing tool by searching for new types of Cas12f or systematically modifying gRNA (Bigelyte et al., 2021; Kim et al., 2021b; Wu et al., 2021). The problem of molecular size has been successfully solved the identification of new compact CRISPR-Cas proteins, and it can be used for the clinical application of gene therapy (Bigelyte et al., 2021; Kim et al., 2021b; Wu et al., 2021). In terms of safety, the subtype III-E effector Cas7-11 can easily cleave and edit RNA without cell toxicity (Özcan et al., 2021). Exploring the diversity of the CRISPR system will result in obtaining editing tools with new functions, such as transposition, to solve a greater number of biological problems.

Although the target site of the genome is cleaved by the CRISPR-Cas system, and then the target DNA fragment can be inserted by homologous or non-homologous recombination, this approach is very inefficient and depends on the characteristics of the cell to be edited. The two editors, BE and PE, can accurately and effectively correct gene mutations, such as point mutations and small genome indels (<60 bp) (Anzalone et al., 2019). However, it is difficult for them to correct large gene alterations (>100 bp). Therefore, efficient and specific integration of target DNA fragments into the genome remains a great challenge. Transposition is a unique genetic recombination that transfers specific genetic factors from one region on the DNA to another. Therefore, transposons can be used to achieve efficient and specific insertion of DNA fragments. Researchers have discovered that some types of CRISPR-Cas systems, such as type I-F and type V-K, are encoded in Tn7-like transposons, which can process and bind crRNA, but cannot cleave target DNA. This suggests that these transposons may be able to mediate RNA-guided site-specific transposition (Peters et al., 2017). Subsequently, experiments have confirmed that transposon-associated CRISPR-Cas systems can mediate RNA-guided site-specific transposition (Klompe et al., 2019; Strecker et al., 2019; Petassi et al., 2020). Through in-depth research on the CRISPR transposition system, the directional insertion of DNA fragments will be realized in the future.

## Conclusion

CRISPR-Cas based toolkits can currently achieve gene knock-out, gene knock-in, single-base substitution, multi-base substitution, and transcription regulation. This technology has greatly accelerated our understanding of biology and disease, and has very strong clinical application prospects for disease treatment. Although the specificity and delivery of gene editing tools currently limit their clinical application, with the increasing amounts of in-depth research that will be conducted in the future, it is likely that gene editing technology will provide effective disease treatment methods and promote the development of the life sciences.

## Glossary

CRISPR: clustered regularly interspaced short palindromic repeats
Cas: CRISPR-associated protein
MGEs: mobile genetic elements
crRNAs: CRISPR RNAs, crRNAs CRISPR RNAs
dsDNA: double stranded DNA
ssRNA: single-stranded RNA
gRNA: guide-RNA
NHEJ: nonhomologous end joining
HDR: homology-directed repair
indels: insertions/deletions
NHP: non-human primate
DETECTR: DNA endonuclease-targeted CRISPR trans reporter
ssDNA: single-stranded DNA
DSB: double-strand break
nCas: Cas nickase
dCas: deactivated Cas
BE: base editor
UGI: glycosylase inhibitor domains
CBEs: cytosine base editors
ABEs: adenine base editors
SNPs: single nucleotide polymorphisms
AAV: adeno-associated virus
PEs: prime editor
pegRNA: prime-editing guide RNA
RNP: ribonucleoprotein
CRISPRi: CRISPR-mediated interference
CRISPRa: CRISPR-mediated activation
LCMV: lymphocytic choriomeningitis virus
IAV: influenza A virus
VSV: vesicular stomatitis virus
SHERLOCK: specific high-sensitivity enzymatic reporter UnLOCKing
REPAIR: RNA editing for programmable A to I replacement
RESCUE: RNA editing for specific C to U exchange
NLS: nuclear localization sequence
ZF: zinc finger
sgRNA: single-guide RNA
PBS: primer binding sequence
RT: reverse transcriptase
